# Effectiveness of a Mobile App to Increase Risk Perception of Tobacco, Alcohol, and Marijuana Use in Mexican High School Students: Quantitative Study

**DOI:** 10.2196/37873

**Published:** 2023-03-09

**Authors:** Patricia María del Carmen Fuentes A, Alberto Jiménez Tapia, Eunice M Ruiz-Cortés, Fernando Bolaños-Ceballos, Julio César Flores Castro, Rafael Gutiérrez, Catalina González-Forteza

**Affiliations:** 1 Direction of Epidemiological and Psychosocial Research National Institute of Psychiatry Ramón de la Fuente Muñiz Mexico City Mexico; 2 Universidad Autónoma del Estado de Hidalgo Actopan, Hidalgo Mexico

**Keywords:** adolescents, students, risk perception, tobacco, alcohol, marijuana, mobile apps, apps, substance use, prevention

## Abstract

**Background:**

Young people have the highest rate of drug use worldwide. Recent data from Mexico in this population show that the prevalence of illicit drug use doubled between 2011 and 2016 (2.9%-6.2%), with marijuana being the one with the highest increase (2.4%-5.3%), but also point out that alcohol and tobacco use have remained steady or decreased. Mexican adolescents are at high risk for drug use owing to a low perception of risk and the availability of drugs. Adolescence is an ideal period to reduce or prevent risky behaviors using evidence-based strategies.

**Objective:**

In this study, we aimed to test the short-term effectiveness of a mobile intervention app (“What Happens if you Go Too Far?” [“¿Qué pasa si te pasas?”]) that seeks to increase risk perception of tobacco, alcohol, and marijuana use in a sample of Mexican high school students.

**Methods:**

A nonexperimental evaluation based on pretest-posttest design was used to measure the effectiveness of a preventive intervention using a mobile app, “What Happens If You Go Too Far?” The dimensions analyzed were knowledge of drugs and their effects, life skills, self-esteem, and risk perception. The intervention was conducted on a high school campus with 356 first-year students.

**Results:**

The sample included 359 first-year high school students (mean 15, SD 0.588 years; women: 224/359, 62.4% men: 135/359, 37.6%). The intervention increased the overall risk perception of tobacco (*χ*^2^_4_=21.6; *P*<.001) and alcohol use (*χ*^2^_4_=15.3; *P*<.001). There was no significant difference in the perception that it is dangerous to smoke 5 cigarettes, and there was a marginal difference in the perception that it is very dangerous to smoke 1 cigarette or to use alcohol or marijuana. We used a generalized estimating equation method to determine the impact of the variables on risk perception. The results showed that knowledge about smoking increased the risk perception of smoking 1 cigarette (odds ratio [OR] 1.1065, 95% CI 1.013-1.120; *P*=.01), and that knowledge about marijuana use (OR 1.109, 95% CI 1.138-1.185; *P*=.002) and self-esteem (OR 1.102, 95% CI 1.007-1.206; *P*=.04) produced significant increases in the risk perception of consuming 5 cigarettes. Resistance to peer pressure and assertiveness also increased the perceived risk of using tobacco and alcohol.

**Conclusions:**

The intervention has the potential to increase the perception of risk toward drug use in high school students by providing knowledge about the effects and psychosocial risks of drug use and by strengthening life skills that are associated with increased risk perception. The use of mobile technologies in intervention processes may broaden the scope of preventive work for adolescents.

## Introduction

### Background

Drug use is shaped by the interaction of personal, social, and contextual factors. Lack of knowledge about substance use dynamics (social influence, positive attitudes toward use, peer group use, availability, and inadequate parental supervision) are elements that favor use in adolescents [1,2]. The most recent data in this population not only show that the prevalence of illicit drug use doubled between 2011 and 2016 (2.9%-6.2%), with marijuana being the one with the highest increase (2.4%-5.3%), but also point out that alcohol and tobacco use have remained steady or decreased [3]. The negative consequences of use affect general well-being, academic performance, physical and mental health, family dynamics, and peer relationships, and they increase the likelihood of fatal accidents [4].

Adolescents and young adults have the highest rates of substance use throughout the world [5]. The data show that adolescents are the population group with the highest risk for substance use in Mexico and that they start at increasingly younger ages. Their perception of risk is low, and drug availability is increasing [6,7]. This situation highlights the need for evidence-based early interventions with a public health approach and an ecological perspective.

During adolescence (10-19 years of age), there are important biological, psychological, and social transitions resulting from rapid physical, psychological, sexual, and social development that involves changes in the brain, cognition, and emotions [1,8]. Adolescents are in a vulnerable position that brings them closer to risk behaviors such as drug abuse [9-12], and it has become a public health problem.

Adolescents are exposed to individual, family, and social risks and protective factors that can increase or decrease the probability of drug use. Family risk factors include conflict, lack of parental supervision, and family members who are abusive physically, emotionally, and sexually. Social risk factors include peer pressure to use drugs as a form of socialization, peers who use it, and the availability of drugs. Individual risk factors include inclination toward experimentation, curiosity, rebelliousness, and impulsivity, as well as low self-esteem, lack of emotional regulation, depression, anxiety, behavioral problems, poor school performance, previous experiences with drugs, and low risk perception toward drug use [13-16]. Risk perception may contribute to increasing the odds of drug use in adolescents, and there is evidence that it may act in both ways as a risk or protective factor; that is, when it is low, it increases the risk of drug use and vice versa [13,16].

Health behavior theories such as the Health Belief Model and the Theory of Planned Behavior suggest that risk perception is an important factor in health behavior and that the level of risk perception determines the likelihood of the occurrence of risk behaviors such as adolescent drug use [14,17-19]. Because risk perception is an attitude that represents the evaluation of an object through its favorable and unfavorable attributes [17], levels of risk perception have been considered important determinants of risky behaviors.

There is evidence indicating that the probability of drug use increases as people perceive little or no risk of associated harm, that is, a higher perceived risk can be considered as a protective factor against drug use [13]. A longitudinal study on risk perception toward tobacco, alcohol, and cannabis use with a 10-year follow-up reported that it reduced the probability of consumption in German adolescents aged 14 and 15 years [18]. Other studies in the Latin American population reported a low risk perception associated with drug use [20], which is usually at these levels for tobacco and alcohol and higher for other drugs. However, a reduction in the perception of risk of marijuana use has also been reported between 2000 and 2014, which has been associated with an increase in the use of this drug in the adolescent population [3].

Evidence shows that a lower perception of risk is related to higher rates of use [21] and that protective dynamics can emerge within the perception of negative consequences or health risks [22-24]. Work is necessary in this regard because the perceived risk of regular marijuana use has decreased by 40% between 1995 and 2019, but the potency of the drug and its consumption have increased; therefore, it is important to reduce this gap to lower its impact on public health [25].

Adolescence is an ideal period for interventions designed to reduce or prevent risky behaviors [26]. Life skills, including positive and adaptive social skills, enable adolescents to cope with everyday challenges [27,28]. The life skills approach considers the following: (1) the recognition and evidence of the role of cognitive, interpersonal, and coping skills in psychosocial development; (2) the effect of skills on young people’s ability to protect their health, adopt positive behaviors, and foster healthy relationships; (3) the application of skills in managing education, violence, and human rights; (4) the reinforcement of protective factors such as self-awareness, self-confidence, and self-esteem; and (5) the mastery and application of skills in everyday situations to feel self-confident, self-efficient, and self-worthy [29,30]. The development of life skills is part of the learning, competence, and education that underpin adolescent well-being [31].

Interventions based on life skills training have proven to be effective for the prevention of drug use in adolescents, are easy to adapt and disseminate [29,32-35], and are an effective strategy to promote health care in schools [27,28,36]. However, their design requires providing knowledge and skills to the professionals who perform them, which in turn requires economic, human, and time resources [8].

Schools are effective settings for preventive interventions, given the ease of access to adolescents and because they are spaces designed to foster learning and socialization [32,37]. Studies on prevention and early intervention based on life skills to address drug use in educational settings have shown excellent results [8]. The COVID-19 pandemic has underscored the importance of measuring the effects of these interventions when they are executed using digital technologies [38].

Mobile apps are effective tools for prevention and intervention in health care. There are various examples of the use of this technology to address depression, anxiety, drug use, and suicide [10,11,39]. Some strategies provide only information and education, and others focus on giving advice, strategies, or skills training, but few offer the possibility of self-assessing drug use and providing feedback [11,39]. Additional research is needed to evaluate the effectiveness of apps, and funding is needed to develop apps using evidence-based techniques [40].

Interventions that are designed for the internet or mobile devices using current evidence-based approaches and resources are more likely to be successful in prevention [11,39,41]. The use of these technologies, including life skills training, has the advantages of accessibility, portability, interactivity, feedback, ease of use, and wide reach at low cost; they reach adolescent and young adult populations because of their ubiquity and mobility and because young people know, accept, and integrate them easily into their lifestyle [32,42].

### Objective

There are few studies that have evaluated the characteristics and effectiveness of digital mobile interventions. It is necessary to evaluate their effectiveness and applicability in real-world contexts to verify their potential and usefulness [32,39-41]. Consequently, the objective of this study was to test the short-term effectiveness of a mobile intervention app (“What Happens if you Go Too Far?” [“¿Qué pasa si te pasas?”]). This study aimed to increase risk perception of tobacco, alcohol, and marijuana use in a sample of Mexican high school students.

## Methods

### Ethics Approval

All participants’ parents signed an informed consent form, and all participants provided written informed consent. The study was approved by the Research Ethics Committee of the Instituto Nacional de Psiquiatría Ramón de la Fuente Muñiz (approval number CEI/C/003/2016).

### Design and Procedure

#### Overview

This study used a nonexperimental pretest-posttest design, with assessments at baseline and at the end of the intervention. The intervention was performed in a high school campus, as part of the introductory curriculum for the school year, in a classroom with 45 computers using the Android emulator BlueStacks (BlueStacks) to execute the app “What Happens if you Go Too Far?” (“¿Qué pasa si te pasas?”). The intervention evaluation questionnaire (Brief Life Skills Scale for Adolescents) (González-Forteza et al, unpublished data, August 2022) was administered as a Google Form. The Brief Life Skills Scale for Adolescents was developed using items from several more extensive scales, each including 35 to 65 items that assess the skills separately and have been validated for Mexican adolescents. These skills include planning for the future, assertiveness, expression of emotions, taking responsibility, decision-making, and resistance to peer pressure.

Before its implementation, teachers and psychology interns received training in the management of the intervention, which included modules on the effects and risks of drug use in adolescents and on strengthening 6 life skills and addressing the emotions that occur when these skills are applied.

The intervention was performed during the orientation week for the incoming students. The students were invited to participate in the study voluntarily and anonymously. The school authorities and teachers were informed of the project and granted access to the school. The objectives of the research as well as the risks and benefits of participating in the study were explained to the students; they were informed that their participation was voluntary, their answers would be anonymous, and the results would not affect their activities or evaluation in school.

In total, 10 groups of first-year high school students from the 2019-2020 classes participated. The intervention lasted 10.5 hours: three 90-minute sessions for each group at school, plus 2 hours per day of individual activities at home using the app during the same week the intervention was implemented. In each of the 3 sessions, there was an average of 36 students per group. Participants did not receive any incentives for their involvement in the study.

#### Session 1

The first session aimed to apply the pretest evaluation, present the mobile app, and explain life skills. The evaluation questionnaire took 20 minutes to complete. After the evaluation, participants were instructed to download the “What Happens If You Go Too Far?” apps on mobile devices (iOS and Android). Downloading the app required students to connect to the internet via a mobile network or Wi-Fi, and once the app was installed on their devices, it could be used offline. The session also included a description of the World Health Organization (WHO)–recommended life skills (decision-making, resistance to peer pressure, problem-solving, future planning, and assertiveness), an explanation of basic emotions, and the link between life skills and the absence or presence of emotions. We also showed the objectives and options included in the main menu of the app and the available resources (comics, video games, quizzes, trivia, agendas, and news). Participants were asked to form teams of 5 to 6 members to share their answers as a group in subsequent sessions on the effects and risks of drug use and the use of life skills in different cases. Finally, we assigned the comic resources on the topics of alcohol consumption and women or men as homework.

#### Session 2

The second session was designed to (1) identify the effects and risks of alcohol use in women and men; (2) apply decision-making skills, resistance to peer pressure, problem-solving, and goal achievement skills; and (3) identify the consequences and emotions associated with the application of the skills. Participants were instructed to read the comic “Alcohol and Women” to understand the risks and immediate effects of alcohol use on women, to use the cases of Mony and Lucy to choose 3 options and their consequences, and to identify the decision-making and resistance to peer pressure involved. Subsequently, they were asked to answer the questions on the “What Happens If You Go Too Far?” app activity form, based on their interaction with the comic to identify the immediate effects of alcohol use and recognize or describe different situations that encourage alcohol use, some of the consequences and emotions involved in using drugs, and some strategies to resist peer pressure. The facilitator asked the teams to answer some questions related to the scenarios described before to reinforce the identification of the immediate social and health effects of alcohol use in women, apply their skills to situations that lead to drug use, associate them with emotions that may occur, and think about the consequences of the characters’ decisions (Lucy and Mony) in the situations they face. Participants then completed the trivia and quiz on alcohol and women to reinforce their knowledge. The second part of the session comprised the same procedure with the comic “Alcohol and Men,” in which problem-solving and goal setting were applied to the cases of the male characters (Beto and Angel). Participants then completed the trivia and quiz on alcohol and men to reinforce their knowledge, and they received orientation on using the comics on tobacco and marijuana to apply negotiation, assertive communication, problem-solving, and resistance to peer pressure.

#### Session 3

The third session sought to (1) identify the effects and risks of tobacco and marijuana use; (2) apply skills related to negotiation, assertive communication, problem-solving, and resistance to peer pressure; (3) identify consequences and emotions associated with the application of the skills; and (4) administer the posttest evaluation questionnaire. Participants were asked to read the comic about tobacco to review the risks and effects of using it. The characters of the comic (Susy, Alma, and Lizet) were used to model the consequences and identify the negotiations and assertive communication involved. Participants then answered the questions on the “What Happens If You Go Too Far?” app activity form to check their understanding of the steps of negotiation, their ability to identify situations that encourage tobacco use, if they could recognize decision-making options and discuss consequences and emotions involved, and if they could apply the life skills in an everyday situation. The facilitator asked the teams to answer some questions related to the scenarios described before to reinforce the identification of the immediate social and health effects of tobacco and marijuana use, apply their skills to situations that lead to drug use, associate them with emotions that may occur, and think about the consequences of the characters’ decisions in the situations they face. Participants then completed the trivia and quiz on tobacco to reinforce their knowledge. The second part of the session followed the same procedure with the comic “Marijuana” to understand the risks and immediate effects of using drugs, and identify the skills involved in problem-solving and resistance to peer pressure involved. This was applied using the cases of the characters (Agus, Diana, Pedro, and Omar). Participants then completed the trivia and quiz on marijuana to reinforce their knowledge. Finally, we administered the posttest evaluation questionnaire (evaluation questionnaire, 20 minutes).

### Intervention to Prevent Addiction: the “What Happens If You Go Too Far?” App

The “What Happens If You Go Too Far?” [43] intervention app seeks to strengthen life skills and increase risk perception of drug use. It is based on the WHO Skills for Health model and Bandura’s Social Learning Theory.

The intervention acts at cognitive and behavioral levels to increase the risk perception of drug use. At the cognitive level, it facilitates the acquisition of specific knowledge of the effects of drugs on the brain and behavior. It contains evidence-based scientific information translated into textual and visual language to generate interactive comics, trivia, quizzes, and games that facilitate understanding and encourage thinking about substance use. At the behavioral level, it strengthens life skills and improves adolescents’ ability to relate with their peers, resist pressure to use substances, solve problems effectively, and make responsible decisions with awareness of consequences. The comics in the app depict and simulate everyday situations related to drug use to apply these skills.

The comics include three elements: (1) information about drugs, their immediate effects, and their risks; (2) situations experienced by young people that are associated with drug use; and (3) skills involved in decision-making, problem-solving, negotiation, assertiveness, and resistance to peer pressure that help to face the challenges of everyday life.

The trivia is a question-and-answer game that facilitates immediate self-assessment of knowledge of the effects of drug use to reinforce the knowledge acquired with the comics. The quiz is a brief, flexible, and self-administered resource based on the WHO alcohol, smoking, and substance involvement screening test, which assesses drug use using 8 questions and indicates low-, moderate-, or high-risk scores [44,45]. It allows participants to identify their individual level of risk and provides them with information to recognize likely consequences and risks.

The video game models situations associated with drug use experienced by the characters Beto and Bety and facilitates the application of skills to making decisions, communicating assertively, negotiating, and resisting peer pressure; it provides automatic reinforcement. The news feature facilitates constant updating of (1) preventive messages or specific events for timely dissemination and (2) links of interest to youth. The agenda feature connects to hotlines that focus on drug use problems.

### The Technology Behind the “What Happens If You Go Too Far?” App

The configuration of comics and game resources uses the potential of interactive technology, using stories that model everyday situations associated with drug use in young people, to practice decision-making with various options and their consequences. This serves to reinforce knowledge and encourage thinking about the risks and effects of drug use.

The interactive dynamic favors (1) reinforcing the knowledge of drugs and their effects, (2) thinking about the risks and consequences of consumption, (3) interactively applying decision-making and its automatic and immediate feedback in the situations presented, (4) concluding reinforcement with infographics of the different skills in each case, and (5) giving immediate feedback on the trivia and quiz. The app content was developed to be both didactic and informative.

The app is available for free on the 2 most important platforms in the market (Google Android [Google LLC] and Apple iOS [Apple Inc]). It can be installed on a wide range of mobile devices and has a broad reach among young people. A third option is to use an Android emulator that allows the app to be used offline on PCs for students and schools without internet access.

The app was developed using Unity 3D and state-of-the-art web technologies. It is based on the current standards of responsive web design and user experience to generate an accessible and optimized product that is fast and easy to download. The front-end offers interactive content and resources that can be used individually or in groups (the game, trivia, and quiz). The information is complemented by an agenda that facilitates contact with specialized care centers and services, as well as links to relevant news and web content. The back end includes various web tools for web-based editing and updating of content, as well as data consultation and statistical reports for use analysis by the developers, administrators, and researchers in charge of the project.

### Data Analysis

In this study, 2 types of analyses were used to identify differences between pre- and posttest measurements. The first was used to measure variations in the perceived risk of using marijuana, alcohol, and tobacco (smoking 1 cigarette and smoking 5 cigarettes) in both measurements. The original categories of risk perception were “not dangerous,” “dangerous,” and “very dangerous.” There was no statistically significant difference between “not dangerous” and “dangerous”; therefore, the former category was excluded from the analysis, and differences in pre- and posttest measurements of risk perception were made between the categories of “dangerous” and “very dangerous.”

Comparisons were performed using McNemar test, with 95% certainty considered statistically significant. This is a nonparametric analysis of the comparison of proportions for 2 related samples whose function is to compare the change in the distribution of proportions between 2 measurements of a dichotomous variable and determine that the difference is not due to chance. In this case, there was no dependent or independent variable, as they were related or paired measurements. The impact of variables on risk perception was calculated using the generalized estimating equation (GEE) method, which extends the generalized linear model to allow for the effect of repeated measurements and other related observations. GEE is a method for modeling longitudinal or pooled data, and is often used with nonnomal data, such as binary or count data. This method uses a set of equations that are solved to obtain parameter estimates. This modeling strategy uses a quasi-likelihood function that assumes only a relationship between μ and Var(Y) rather than a specific distribution for Y. This allows deviation from the usual assumptions, such as overdispersion caused by correlated observations or unobserved explanatory variables. To do this, the quasi-likelihood approach takes the usual formula for variance but multiplies it by a constant that is estimated using the data. GEE is designed for simple clustering or repeated measures; it is not easily adaptable to more complex designs, such as nested or cross-group designs [46].

We took care in the analysis that (1) the mean structure was correctly specified (all relevant variables were included and all irrelevant variables were removed), (2) the observations between clusters were unrelated (there is no higher-level clustering mechanism), (3) the sample size was large enough for asymptotic inference (356 records); (4) the normality of residuals was not assumed with GEE; and (5) the database was restructured to obtain the necessary information. As there is no field called TIME, we used the SAMPLE field, which in our case, expresses the ratio of time between evaluations as a function of TIME. Although it was designed for longitudinal studies, in this case, it was applied to 2 measurements because missing measurements are common in longitudinal designs and are assumed to be caused by chance. Therefore, missing values were imputed using IBM SPSS Modeler (version 18.3; IBM Corp) tool, which has different imputation methods (fixed, random, expression, and algorithm). In this analysis, we used the algorithm method, which replaces a predicted value with a model based on the classification and regression trees algorithm. In each field imputed with this method, there is a separate classification and regression trees model, along with a fill node that replaces empty and null values with the value predicted by the model.

## Results

### Participants

The sample for the initial evaluation included 359 (90% of the 399 enrolled students) first-year middle-class high school students at the Escuela Superior Actopan, affiliated with the Universidad Autónoma del Estado de Hidalgo. The mean age of the participants was 15 (SD 0.588) years. Of the total sample, 224 (62.4%) of the students were women and 135 (37.6%) were men; 182 (50.7%) participants were in the morning session and 176 (49%) participants were in the afternoon session. Regarding drug use history, 64 (17.8%) participants reported tobacco use sometime in life (women: 36/224, 16.1%; men: 28/135, 20.7%) and 27 (7.5%) in the past 3 months (women: 11/224,4.9%; men: 16/135, 11.9%); 174 (48.5%) students reported alcohol use sometime in life (women: 99/224, 44.2%; men: 75/135, 55.6%) and 126 (35.1%) in the past 3 months (women: 81/224, 36.2%; men: 45/135, 33.3%); 18 (5%) participants reported marijuana use sometime in life (women: 7/224, 3.1%; men: 11/135, 8.1%) and 6 (1.7%) in the past 3 months (women: 1/224, 0.4%; men: 5/135, 3.7%; Multimedia Appendix 1).

The follow-up evaluations were completed by 356 (99.2%) of the 359 study participants. The mean age of the participants was 15 (SD 0.574) years. Of them, 224 (62.9%) participants were women, 132 (37.1%) were men; 224 (62.9%) were in the morning session, and 132 (37.1%) were in the afternoon session. The inclusion criterion was enrollment in the first semester of high school at Escuela Superior Actopan.

### Effectiveness of the App

Of the 356 students, the proportion of those who perceived that it was dangerous to smoke 1 cigarette decreased from 197 (55.3%) to 161 (46%) comparing the pre- and posttest measurements, but the perception that it was very dangerous increased from 85 (23.9%) to 142 (39.9%). Of the 356 students, the proportion perceiving that it was dangerous to smoke 5 cigarettes increased from 62 (17.4%) to 73 (20.5%) comparing the pre- and posttest measurements, and the perception that it was very dangerous decreased from 288 (80.9%) to 271 (76.1%).

The proportion perceiving that it was dangerous to consume alcohol decreased from (193/356, 54.2%) to (131/356, 36.8%) comparing the pre- and posttest measurements, and the perception that it was very dangerous increased from (156/356, 43.8%) to (210/356, 60%).

Similarly, the proportion of the students perceiving that it was dangerous to use marijuana decreased from (141/356, 39.6%) to (111/356, 31.2%) and that it was very dangerous increased from (203/356, 57%) to (235/356, 66%).

The overall risk perception of the use of these drugs showed significant differences only for the use of 1 cigarette (*χ*^2^_4_=21.6; *P*<.001) and the use of alcohol (*χ*^2^_4_=15.3; *P*<.001); there was a marginal difference for the use of marijuana (*χ*^2^_4_=3.6; *P*=.057). There was no significant difference in the risk perception of smoking the 5 cigarettes.

Tables 1-4 show the GEE models in which gender, drug knowledge, life skills, and self-esteem were included as covariates (Multimedia Appendix 2). The analysis showed that knowledge of smoking was the variable that generated a significant increase in risk perception (odds ratio [OR] 80.060, 95% CI 0.009-0.110; *P*=.02) of smoking 1 cigarette (Table 1).

Knowledge of marijuana (OR 0.091, 95% CI 0.023-,0.160; *P*=.009), and self-esteem (OR 0.131, 95% CI 0.046-0.217; *P*=.003), were the variables that produced significant increases in the risk perception of consuming 5 cigarettes (Table 2).

The variables that increased the risk perception of alcohol consumption were knowledge of smoking (OR 0.089, 95% CI 0.043-0.135; *P*<.001), assertiveness (OR 0.102, 95% CI 0.004-0.200; *P*=.04), resistance to peer pressure (OR 0.078, 95% CI 0.023-0.134; *P*=.006), and self-esteem (OR 0.140, 95% CI 0.063-0.216; *P*<.001; Table 3).

For perceived risk of marijuana use, the variables that significantly increased risk perception were knowledge of tobacco (OR 0.074, 95% CI 0.026-0.121; *P*=.002), knowledge of marijuana (OR 0.110, 95% CI 0.047-0.174; *P*=.001), resistance to peer pressure (OR 0.117, 95% CI 0.060-0.174; *P*<.001), and self-esteem (OR 0.165, 95% CI 0.085-0.244; *P*<.001) (Table 4).

**Table 1:** Generalized estimating equation model for the risk perception of smoking one cigarette.

Variable	Exp(B)	95% Wald CI for exp(B)	*P* value
(Intersection)	−6.555	−8.725 to −4.385	<.001
Gender (men)	0.017	−0.323 to 0.357	.92
Knowledge about tobacco	0.060	0.009 to 0.110	.02
Knowledge about marijuana	0.038	−0.030 to 0.106	.28
Knowledge about alcohol	0.002	−0.030 to 0.034	.90
Planning for the future	−0.015	−0.090 to 0.059	.68
Assertiveness	0.083	−0.023 to 0.189	.12
Expression of emotions	0.069	−0.024 to 0.162	.14
Resistance to peer pressure	0.003	−0.057 to 0.062	.93
Decision-making	0.023	−0.035 to 0.080	.44
Taking responsibility	0.015	−0.115 to 0.145	.82
Self-esteem	0.059	−0.025 to 0.142	.17

**Table 2:** Generalized estimating equation model for the risk perception of smoking 5 cigarettes.

Variable	Exp(B)	95% Wald CI for exp(B)	*P* value
(Intersection)	−3.265	−5.452 to −1.078	.003
Gender (men)	0.132	−0.255 to 0.518	.50
Knowledge of tobacco	0.023	−0.029 to 0.075	.39
Knowledge of marijuana	0.091	0.023 to 0.160	.009
Knowledge of alcohol	−0.006	−0.042 to 0.031	.77
Planning for the future	−0.062	−0.157 to 0.032	.20
Assertiveness	0.077	−0.039 to 0.193	.20
Expression of emotions	0.007	−0.096 to 0.110	.89
Resistance to peer pressure	0.027	−0.035 to 0.090	.39
Decision-making	−0.024	−0.087 to 0.039	.46
Taking responsibility	−0.028	−0.175 to 0.118	.70
Self-esteem	0.131	0.046 to 0.217	.003

**Table 3:** Generalized estimating equation model for the risk perception of frequent alcohol use.

Variable	Exp(B)	95% Wald CI for exp(B)	*P* value
(Intersection)	−8.326	−10.404 to −6.248	<.001
Gender (men)	0.087	−0.238 to 0.412	.60
Knowledge of tobacco	0.089	0.043 to 0.135	<.001
Knowledge of marijuana	0.005	−0.056 to 0.066	.87
Knowledge of alcohol	0.010	−0.020 to 0.041	.50
Planning for the future	−0.047	−0.143 to 0.049	.34
Assertiveness	0.102	0.004 to 0.200	.04
Expression of emotions	0.019	−0.068 to 0.106	.66
Resistance to peer pressure	0.078	0.023 to 0.134	.006
Decision-making	0.020	−0.033 to 0.073	.46
Taking responsibility	0.062	−0.060 to 0.183	.32
Self-esteem	0.140	0.063 to 0.216	<.001

**Table 4:** Generalized estimating equation model for the risk perception of marijuana use.

Variable	Exp(B)	95% Wald CI for exp(B)	*P* value
(Intersection)	−9.428	−11.611 to −7.246	<.001
Gender (men)	0.314	−0.030 to 0.658	.07
Knowledge of tobacco	0.074	0.026 to 0.121	.002
Knowledge of marijuana	0.110	0.047 to 0.174	.001
Knowledge of alcohol	−0.011	−0.044 to 0.022	.51
Planning for the future	−0.040	−0.106 to 0.026	.23
Assertiveness	0.045	−0.059 to 0.149	.40
Expression of emotions	−0.033	−0.126 to 0.061	.49
Resistance to peer pressure	0.117	0.060 to 0.174	<.001
Decision-making	0.049	−0.007 to 0.105	.08
Taking responsibility	−0.025	−0.153 to 0.104	.71
Self-esteem	0.165	0.085 to 0.244	<.001

## Discussion

### Principal Findings

The objective of this study was to evaluate the effectiveness of a mobile app in increasing the risk perception of tobacco, alcohol, and marijuana use based on life skills and self-esteem training components in a sample of Mexican high school students. The results of the study showed four main findings regarding the intervention: (1) it increased the overall risk perception of tobacco and alcohol use, (2) it increased the perception that it was dangerous to smoke 5 cigarettes, (3) it increased the perception that it was very dangerous to smoke 1 cigarette or to use alcohol or marijuana, and (4) the variables that significantly increased the risk perception of using these drugs were knowledge of tobacco, knowledge of marijuana, resistance to peer pressure, assertiveness, and self-esteem.

Risk perception of the possible consequences of drug use is usually low in adolescents and young adults [47], and evidence has shown that this low perception of risk can increase the likelihood of using drugs [48]. Risk perception is also associated with the type and knowledge of drugs [49]. For example, the recreational use of marijuana in Mexico is still not legal. Although the Supreme Court voted in favor of personal possession and consumption for recreational purposes in 2022, the stigma surrounding the use of this substance is still considerable, which could have an important impact on the perception of risk toward its use.

Interventions that are designed to increase risk perception have the potential to be useful tools in developing strategies to address drug use in this population. Interventions involving life skills training have shown satisfactory effects in reducing drug use and on attitudes and beliefs toward drug use [38]. It is therefore plausible to think that life skills education could also have a positive effect on risk perception and help reduce the likelihood of drug use.

Evidence shows that life skills education helps modify the risk perception of potentially harmful behaviors [50] and drug use [51].

Our results showed that the self-esteem component of the intervention significantly increased the risk perception of tobacco, alcohol, and marijuana use. This finding should be viewed with caution, given that the evidence on the relationship between self-esteem and the perception and display of risky behaviors is inconsistent. Some studies have reported weak to moderate relationships, while others have reported no correlation between these constructs [52]. However, there is also evidence that self-esteem is a protective element against potentially dangerous behaviors [53-57]. Despite the controversy in the data and in the conceptualization of self-esteem, we believe that its inclusion in intervention programs that seek to reduce substance use could be an important way to improve the results because it has been found to be relevant in improving life skills that contribute to reducing exposure to risk factors and improving the ability to protect themselves from situations that adolescents experience daily [58,59].

### Limitations

The results of this study should be considered in light of its limitations. It is necessary to explore the reasons why there was no significant difference in the perception of risk between not dangerous and dangerous and to design strategies to achieve change in these categories. Another limitation is that no variables that could moderate the effect of the modification of risk perception were identified; therefore, the impact of these variables is unknown. We are not unaware of the possible influence of threats to the internal validity of this study. For example, it is possible that there were some events between the 2 measurements that could have influenced the results and of which we had no record. In addition, the selection of the participants could have had some effect, that is, because they were new students to a prestigious school, they could have responded orientated by social desirability and by certain knowledge of or familiarity with the measurement instrument. These threats can be minimized in future studies using quasi-experimental or true experimental designs. It is also important to consider the conditions for the implementation of the intervention, for example, the optimal institutional conditions of technological infrastructure, the selection and training of personnel to administer the intervention, and its incorporation into the school program as a support tool in the curriculum. Another limitation is that given the sample selection strategy, the results are not generalizable to the entire adolescent population. We also acknowledge that our intervention is very brief and this may pose a threat for effectiveness, but our results provide evidence of the potential of this intervention to become a cost-effective tool for prevention strategies and for continuing work on the impact of life skills in modifying risk perception and for the evaluation of this type of intervention in longitudinal studies. Another important element to consider is that it would be very enriching to include more detailed measures of risk perception that would allow an exhaustive taxonomy for the analysis, for example, the perception of risk of possible harm and of sanctions or the risk related to stigma and the emotional risk associated with drug use.

### Conclusions and Perspectives

The development of the “What Happens If You Go Too Far?” app focused on increasing the risk perception of substance use in adolescents, based on the evidence that this perception may reduce the probability of use [16] and that the perception of negative health consequences can generate protective dynamics [6,22,24,48]. It is important to note that more research using more robust designs and sampling strategies is necessary. Our results show that the app has the potential to increase the risk perception of alcohol, tobacco, and marijuana use in high school students, and by providing evidence-based content on the psychosocial effects and risks of the use of each of these drugs and strengthening of life skills (assertiveness and resistance to peer pressure), which were significantly associated with increased risk perception, it would be plausible to improve the effectiveness of interventions aimed at preventing drug use in adolescents and youth.

The app is an accessible resource that can be included in intervention strategies for prevention, as it strengthens life skills that are useful as self-care strategies for adolescents and favors prevention with different social actors, such as educational and health centers that interact with this population, using basic technological resources. The app may also become popular in a youth population that has accepted and integrated mobile technologies into its lifestyle [8,42], thanks to their accessibility, usability, portability, interactivity, and ease of use. It provides a broad reach at low cost, with the likelihood of expansion among the adolescent population.

The incorporation of mobile technologies as tools to reduce drug use or delay the onset of drug use can be fundamental in reaching larger numbers of people with knowledge of the most commonly used drugs that are considered gateway substances to more serious drugs [8,25,41].

## Appendix

Multimedia Appendix 1 Substance use history pre-intervention.

Multimedia Appendix 2 Code for the analyses.

## Abbreviations

GEE: generalized estimating equation
OR: odds ratio
WHO: World Health Organization
