# Supplementary material for: Effectiveness of a Mobile App to Increase Risk Perception of Tobacco, Alcohol, and Marijuana Use in Mexican High School Students: Quantitative Study
Source: JMIR Mhealth Uhealth. 2023 Mar 9;11:e37873. doi: 10.2196/37873 (PMC10037168; doi:10.2196/37873)
Supplement: Multimedia Appendix 1 [file mhealth_v11i1e37873_app1.pdf]

Substance use history pre-intervention (N=359)

Drug use	Women (n=224)		Men (n=135)		Total	
	Sometime in life	Sometime past three months	Sometime in life	Sometime past three months	Sometime in life	Sometime past three months
Tobacco	36 (16.1%)	11 (4.9%)	28 (21.1%)	16 (12.2%)	64 (17.8%)	27 (7.5%)
Alcohol	99 (44.2%)	81 (36.1%)	75 (56.4%)	45 (34.1%)	174 (48.5%)	126 (35.1%)
Marijuana	7 (3.1%)	1 (0.4%)	11 (8.3%)	5 (3.8%)	18 (5.0%)	6 (1.2%)
