# Supplementary material for: Effectiveness of a Mobile App to Increase Risk Perception of Tobacco, Alcohol, and Marijuana Use in Mexican High School Students: Quantitative Study
Source: JMIR Mhealth Uhealth. 2023 Mar 9;11:e37873. doi: 10.2196/37873 (PMC10037168; doi:10.2196/37873)
Supplement: Multimedia Appendix 2 [file mhealth_v11i1e37873_app2.doc]

Appendix 2

Code for the analyses

**GENLIN s7a_r (REFERENCE=LAST) BY sexo (ORDER=ASCENDING) WITH Conoc_Tabaco Conoc_Mariguana**

**Conoc_Alcohol PlaneaciónFuturo Asertividad ExprEmociones PresiónPares TomaDecisiones**

**TomaResponsabilidades Autoestima**

**/MODEL sexo Conoc_Tabaco Conoc_Mariguana Conoc_Alcohol PlaneaciónFuturo Asertividad ExprEmociones**

**PresiónPares TomaDecisiones TomaResponsabilidades Autoestima INTERCEPT=YES**

**DISTRIBUTION=BINOMIAL LINK=LOGIT**

**/CRITERIA METHOD=FISHER(1) SCALE=1 MAXITERATIONS=100 MAXSTEPHALVING=5 PCONVERGE=1E-006(ABSOLUTE)**

**SINGULAR=1E-012 ANALYSISTYPE=3(WALD) CILEVEL=95 LIKELIHOOD=FULL**

**/REPEATED SUBJECT=fgralpre WITHINSUBJECT=Muestra SORT=YES CORRTYPE=UNSTRUCTURED ADJUSTCORR=YES**

**COVB=ROBUST MAXITERATIONS=100 PCONVERGE=1e-006(ABSOLUTE) UPDATECORR=1**

**/MISSING CLASSMISSING=EXCLUDE**

**/PRINT CPS DESCRIPTIVES MODELINFO FIT SUMMARY SOLUTION.**
